# Communication between mitochondria and other organelles: a brand-new perspective on mitochondria in cancer

**DOI:** 10.1186/s13578-019-0289-8

**Published:** 2019-03-19

**Authors:** MengFang Xia, YaZhuo Zhang, Ke Jin, ZiTong Lu, Zhaoyang Zeng, Wei Xiong

**Affiliations:** 1 NHC Key Laboratory of Carcinogenesis, Xiangya Hospital, Central South University, Changsha, Hunan China; 2 The Key Laboratory of Carcinogenesis and Cancer Invasion of the Chinese Ministry of Education, Cancer Research Institute, Central South University, Changsha, Hunan China; 3 Hunan Key Laboratory of Non Resolving Inflammation and Cancer, Disease Genome Research Center, The Third Xiangya Hospital, Central South University, Changsha, Hunan China

**Keywords:** Organelle communication, Mitochondria, Endoplasmic reticulum, Peroxisomes, Cell nucleus, Carcinogenesis

## Abstract

Mitochondria are energy factories of cells and are important pivots for intracellular interactions with other organelles. They interact with the endoplasmic reticulum, peroxisomes, and nucleus through signal transduction, vesicle transport, and membrane contact sites to regulate energy metabolism, biosynthesis, immune response, and cell turnover. However, when the communication between organelles fails and the mitochondria are dysfunctional, it may induce tumorigenesis. In this review, we elaborate on how mitochondria interact with the endoplasmic reticulum, peroxisomes, and cell nuclei, as well as the relation between organelle communication and tumor development 
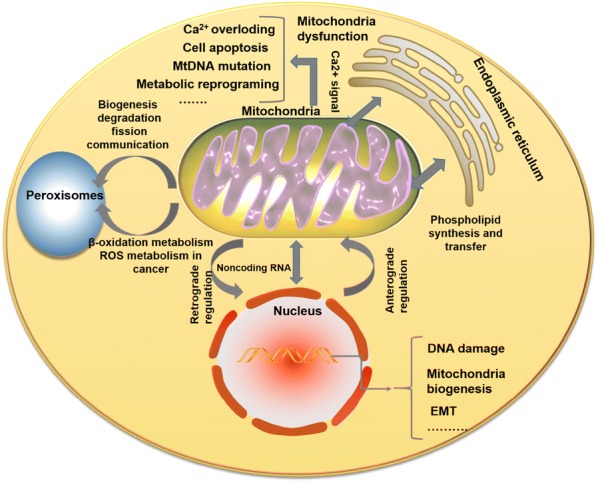
.

## Introduction

The mitochondrion is a double membrane-bound half-independent organelle that shoulders most of the metabolic activities in the cell. The mitochondria modulate cell metabolism, reactive oxygen species (ROS) genesis, cell apoptosis, and the universal second messenger, calcium [1].

Mitochondria, as energy centers, play an important role in cancer metabolism. The first description of the function of mitochondria in tumorigenesis used the term ‘aerobic glycolysis’, which is now known as the Warburg effect [2]. This effect is based on the fact that tumor cells use glycolysis rather than the tricarboxylic acid (TCA) cycle for energy production. Warburg attributed this phenomenon to mitochondrial dysfunction in tumor cells and suggested that destruction of the mitochondrial respiratory chain caused tumor cells to rely on glycolysis. However, mitochondrial dysfunction is not considered a hallmark of cancer, as it also correlated with other diseases such as heart failure, diabetes, hepatocerebral disorder, juvenile catastrophic epilepsy, and neurodegenerative diseases [3–5]. Additionally, tumor cells require fully functional mitochondria. The conditions of tumor cells are quite different from those of normal cells; in fact, tumor cells develop new mechanisms to adapt to these changes.

Meanwhile, as an important organelle, the stability of the mitochondria requires not only its own regulation but also a finely tuned interplay with other organelles [4]. These organelles constitute a complicated network, and the dysregulation of one of the downstream pathways may lead to severe mitochondrial dysfunction, resulting in their failure to regulate energy metabolism and ion buffering.

The crosstalk between mitochondria and other organelles is important in tumorigenesis; because the organelles work as an entity, any impairment in the relevant cascades may lead to change in cell microenvironment, activation of certain oncogenes, and mitochondrial genome mutation. This review introduces the connections between the mitochondria and endoplasmic reticulum (ER), mitochondria and peroxisome, and mitochondria and nucleus in physiological and pathological conditions, as well as the impact of this crosstalk in cancer pathogenesis.

## Mitochondria and ER

In the past few decades, several studies have shown that mitochondria are widely associated with the ER. There are many ways in which the ER can interact with the mitochondria. However, the most important way is through their membrane structures, which contact each other but do not fuse; so, they retain their individual characteristics. The area of close contact between the mitochondria and the ER membrane can be observed in animal cells by electron microscopy and fluorescence microscopy. The contact point is 10–30 nm wide, where it can attach to ribosomes [6]. The contact points between the mitochondria and the ER are relatively stable. When the ER and mitochondria move along the cytoskeleton, the two organelles maintain contact with each other.

**Fig. 1:**
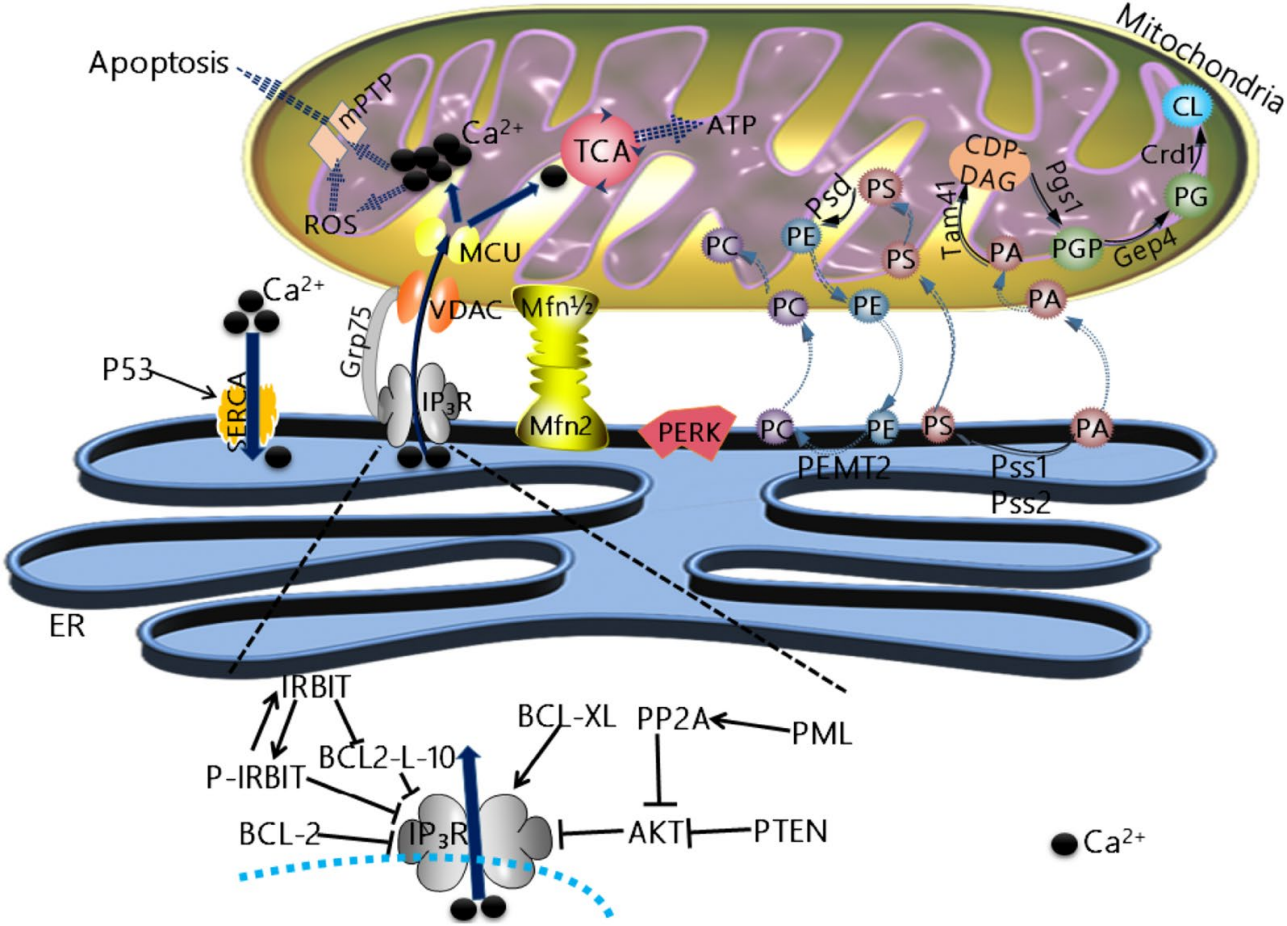
The interaction between mitochondria and ER. Calcium is released via inositol 1,4,5 trisphosphate receptor (IP3R) of the endoplasmic reticulum (ER) and provides a relatively high calcium environment for mitochondria. Calcium enters the mitochondrial matrix through the voltage-dependent anion channels (VDACs) on the outer mitochondrial membrane (OMM) and through the low-affinity receptor, mitochondrial calcium uniporter (MCU) on the inner mitochondrial membrane (IMM). The glucose-regulated protein 75 (GRP-75) can be used as a partner to connect IP3R to VDACs. In addition, IP3R is regulated by numerous regulatory mechanisms. Mitofusin 2 (Mfn-2) on the ER interacts with Mfn-1 or Mfn-2 on the OMMs and regulates the connection between the two organelles. After lipid synthesis in the mitochondria or ER, a large amount of lipids is exchanged between the mitochondria and the ER in order to achieve the final lipid composition of the two organelles. This includes the processes of phosphatidylethanolamine (PE) and phosphatidylcholine (PC) synthesis and cardiolipin (CL) synthesis

Relatively stable contacts provide the basis for the interaction between ER and mitochondria to coordinate cellular biological functions, such as calcium ion (calcium) signaling, apoptosis regulation, ER stress response, phospholipid synthesis, and translocation of the phospholipid from the ER membrane to the mitochondrial membrane. These contact sites are called mitochondria-associated ER membranes (MAMs) (Fig. 1). MAMs are rich in calcium transport channels, enzymes for lipid synthesis and transport and proteins encoded by oncogenes that regulate cellular signaling pathways, and tumor suppressors. Therefore, changes in the above mechanisms may be related to the occurrence and development of cancer. The protein on MAM may be involved in tumorigenesis and tumor progression (Table 1).

**Table 1:** MAMs proteins involved in tumorigenesis and tumor progression

Protein	Gene expression in cancer	MAM interactors	Functions at MAM	Chemotherapeutic agents that act through mechanisms related to the MAMs	References
AKT	Pancreas (A, D), breast (M), prostate (A)	IP3R, PTEN, PML	Inhibition of calcium release from the ER; antiapoptotic functions	MK-2206	[22, 25, 39–41]
BCL-XL	Uterus (A, M), breast (A), prostate (A), colon (A), nervous system (D, M)	BCL-2, IP3R	Induction of calcium leakage from ER; antiapoptotic functions	ABT-737	[30, 42–45]
BCL-2	B cell (M), central nervous system (A, M), pancreas (D), breast (A)	BCL-XL, IP3R	Induction of calcium leakage from ER; antiapoptotic functions	ABT-737	[31, 43–46]
MFN-2	Pancreas (A, D), esophagus (M, A, D), prostate (M, A, D)	PERK, MFN-1	Facilitates calcium cross-talk between the ER and mitochondria;Interacts with PERK and regulates PERK-mediated UPR		[47–49]
PML	Almost all	AKT, IP3R, PP2A	Regulates apoptosis in the ER by modulating calcium release, negative regulator of Akt	Arsenic trioxide	[24, 27, 50]
PP2A	Prostate (A), central nervous system (M), pancreas (A, D)	AKT, PML	Regulates calcium transients in cardiomyocytes	SMAPs	[51–53]
PTEN	Uterus (M), prostate (M, D), head (M, D), stomach (M), breast (A, M), pancreas (M)	AKT, IP3R, PP2A	Negative regulator of Akt, regulation of calcium release via IP3R3; proapoptotic functions	LY-2779964	[54–56]
TP53	Almost all	SERCA	Interacts with the C-terminal portion of the SERCA pump, increasing ER Ca2+ loading	Adriamycin	[57–59]
PERK	Breast (A)		Involved in folded protein response during ER stress; physically increases contacts between mitochondria and ER	GSK2656157/GSK2606414	[60–62]

*A* Amplification, *M* mutation, *D* deletion

### Calcium signal and role in the communication between mitochondria and ER

Calcium is a very important intracellular regulatory molecule. It regulates a variety of physiological and pathological processes, including cancer, and an increasing number of studies have shown that oncogenes and tumor suppressors are also related to the calcium transport system (Fig. 1).

Mitochondria and ER are important storage organelles of calcium in the cell, and calcium transfer between organelles is crucial for cell life and death [7, 8]. Calcium enters the mitochondria from the ER through MAMs and plays an important role in mitochondrial division and control of apoptosis. The uptake of calcium in the mitochondrial matrix is mainly accomplished by the low-affinity mitochondrial calcium uniporter (MCU) receptor on the inner mitochondrial membrane (IMM), and calcium passes through the outer mitochondrial membrane (OMM) relatively easily, mainly via the voltage-dependent anion channels (VDACs) [9–11]. Therefore, to promote the efficient uptake of calcium by mitochondria, it is necessary to generate locally high concentrations of calcium in MAMs. MAMs are highly enriched in the sensitive calcium channel’s inositol 1,4,5 trisphosphate receptor (IP3R). Under the action of IP3R and other signals, calcium in the ER is rapidly released into the surrounding cytoplasm through IP3R, exposing mitochondria to higher concentrations of calcium [12, 13].

Calcium transfer can be terminated by increasing the distance of MAMs [13, 14]. In mammalian cells, mitofusin 2 (MFN2), a family member of mitochondrial dynamics, is located in the OMM and ER surface, regulating the organelle connection between calcium-transfer sites [15–17]. ER-resident MFN2 interacts with mitochondrial mitofusin 1 (MFN1) and MFN2. Efficient IP3R-mediated calcium transfer to the mitochondria is achieved by the chaperone protein, a 75-kDa glucose regulatory protein (GRP75). This protein physically links the VDAC channel on the mitochondrial outer membrane to IP3R on the ER in MAMs. Knockout of GRP75 impairs IP3R-mediated transfer of calcium to the mitochondria [18].

However, this sensitive system can be utilized to achieve malignant transformation of cells. Several types of cancer cells undergo extensive reorganization of calcium signaling mechanisms to become conducive to tumorigenesis [19].

The cancer cells have altered calcium regulation mechanisms involving IP3R and VDAC, enabling the survival of cancer cells [19]. Additionally, the presence of proteins encoded by oncogenes and tumor suppressors in MAMs may alter calcium signaling in cancer cells.

Recent studies showed that disturbance in calcium homeostasis is an important mechanism of oncogene-encoded proteins and tumor suppressors to affect cancer cell fate [20]. Because IP3R is an important calcium transport system that maintains calcium homeostasis between the ER and mitochondria, some oncogene-encoded proteins and tumor suppressors have been shown to modulate IP3R activity. Therefore, IP3R is considered a molecular target for the action of oncogene-encoded proteins and tumor suppressor factors in cancer cells (Fig. 1).

IP3R is regulated by a variety of mechanisms. IP3R on the MAMs can serve as signal centers, and multiple signals can act on them. Different signals are brought together and converted to calcium signals, further affecting the function of mitochondria and even cells [21]. IP3R is over-phosphorylated in cancer cells upregulated by AKT [22]; therefore, calcium flow from the ER to mitochondria decreases, which protects cancer cells from mitochondrion-mediated apoptosis.

Thus, phosphorylated IP3R is regulated by numerous different oncogene-encoded proteins and tumor suppressors, including AKTs lipid phosphatase and negative regulators, phosphatase and tensin homolog on chromosome 10 (PTEN) [23], p53 proteins [24], and promyelocytic leukemia (PML) proteins. Studies showed that PML in MAMs is part of a complex composed of AKT. PML weakens the ability of PP2A to bind to IP3R, and PP2A does not dephosphorylate to inactivate AKT. This leads to phosphorylation of AKT, which phosphorylates IP3R and inhibits the release of calcium from protein IP3R, further protecting the mitochondria from calcium-mediated apoptosis [25]. PML is an effective tumor suppressor protein that stabilizes p53 protein and improves its function. Deletion of PML is associated with malignant pleural mesothelioma, breast cancer, etc. [26, 27]. Additionally, PML inhibits the formation of autophagosomes, thereby inhibiting autophagy induction. Decreased PML levels can also promote tumor growth by enhancing cellular autophagy [24]. Some anticancer drugs target the calcium-connected pathways [28]. For example, arsenic trioxide has a significant effect on chemotherapy for acute promyelocytic leukemia by specifically targeting PML. This chemotherapy increases the level of PML in MAMs and increases IP3R-mediated calcium transfer between the ER and mitochondria, thereby promoting the apoptosis of tumor cells and reducing autophagy [24] (Table 1).

Bcl-2 family proteins in the ER play an important role in apoptosis by controlling the integrity of mitochondrial membrane, the release of cytochrome C, and the activation of caspases [29]. It has been reported that Bcl-2 family proteins regulate the activity of IP3R [30], and different Bcl-2 proteins bind to IP3R at different sites and play different roles (Table 1). For example, Bcl-2 binds to the central region of IP3R, thereby inhibiting the function of IP3R and reducing the release of calcium, which leads to the inhibition of the apoptotic signal. Besides, BCL-XL interacts with the most C-terminal region of IP3R to promote calcium entry into the mitochondria [31]. Studies have shown that the BH4-domain of BCL-XL can selectively target and inhibit the n-terminal domain of VDAC1, while the BH4 domain of Bcl-2 is the only one involved in the inhibition of IP3Rs [32]. In addition, the interaction between Bcl-2-like protein 10 (Bcl2-L-10), a member of the anti-apoptotic Bcl-2 family of proteins, and IP3Rs-binding protein released with IP3 (IRBIT), regulates the activity of IP3R. IRBIT antagonizes Bcl2-L-10, with both proteins binding to the IP3-binding domain of IP3R1. When IRBIT and Bcl2-L-10 complexes are phosphorylated, they bind to IP3R and inhibit its activity, reducing calcium release. Under the stimulation of apoptosis, IRBIT and Bcl2-L-10 complex is dephosphorylated, separated from IP3R, and calcium is released by IP3R, resulting in calcium flowing into the mitochondria, leading to apoptosis [33].

Additionally, the sarco/endoplasmic reticulum calcium ATPase (SERCA) pump localizes to the ER membrane. It is regulated by several proteins at the ER-mitochondrial contact site to affect calcium flux. The SERCA pump ensures refilling of the ER calcium storage by actively pumping calcium ions from the cytosol to the ER, creating a high calcium gradient between these regions (~ 0.1 μM in the cytosol and ~ 400 μM in the ER) [34]. The SERCA2b subtype is the most abundant in MAMs [35]. Regulation of the activity of the SERCA pump by proteins encoded by oncogenes in MAMs and tumor suppressor factors is also important for the development of tumor cells. For example, the p53 protein in MAMs can maintain the activity of the SERCA pump, which is beneficial for maintaining the calcium ion concentration in the ER. When apoptosis is triggered, the ER releases a large number of calcium ions, which enter the mitochondria to cause a calcium ion overload and induce apoptosis. However, in cancer cells, TP53 may be mutated or the p53 protein is inactivated, and thus, the ER cannot maintain a state of relatively high calcium ions, enabling cancer cells to escape apoptosis (Fig. 1).

Stromal interaction molecule 1 (STIM1) is an important calcium sensor located in the ER, which activates ORAI store-operated calcium entry (SOCE) channels. SOCE affects ER calcium content through the activity of calcium-released activated channels (CRAC). CRAC channels are mainly composed of ORAI proteins, which are activated by the ER calcium sensors, stromal interacting proteins or STIMs [36]. In cancer, STIM and ORAI isoforms display increased expression in numerous tumor types and are associated with signaling pathways that positively regulate cancer cell proliferation, migration, invasion, and chemoresistance [37, 38].

### Mitochondria and ER stress response

The response of ER to cellular stress is linked to the accumulation of unfolded proteins and called unfolded protein response (UPR). UPR is activated in response to the accumulation of unfolded or misfolded proteins accumulated in the ER. UPR stops protein translation, degrades misfolded proteins, and activates signaling pathways to restore the normal function of cells. As a large number of molecular chaperones assist in the folding of unfolded proteins, they consume a large amount of ATP. Therefore, in order to increase the production of ATP, cells usually increase the contact area between ER and mitochondria, which in turn increases the release of calcium from the ER, causing calcium to flow into mitochondria [63]. If UPR does not reduce cell stress, the ER and mitochondrial contact points resulting from the above process increase, calcium release increases, and mitochondria uptake calcium, leading to apoptosis (Fig. 1).

In cancer cells, UPR is constitutively activated. During tumor development and growth, abnormal cell proliferation requires higher protein synthesis, and cancer cells are subjected to various pressures such as hypoxia, low glucose, low pH, and lack of nutrition, which induce UPR [64]. UPR signaling is initiated by its three mediators: RNA-dependent protein kinase-like kinase (PERK), activating transcription factor 6 (ATF6), and inositol-requiring enzyme 1α (IRE1α) [65].

PERK in MAMs and MAM-resident PERK were shown to have heterogeneous functions (Fig. 1, Table 1). Hence, only PERK is described below. Activation of PERK signaling and integrated stress response (ISR) is considered a necessary condition for tumor survival under conditions of hypoxia and nutrient deficiency [66]. The activated PERK pathway phosphorylates eukaryotic translation initiation factor 2α (eIF2α). Phosphorylation of eIF2 can be induced by a variety of kinases, including protein kinase R, general control non-repressed 2, and heme-regulated eIF2α kinase. EIF2α phosphorylation-related signaling is described as the ISR. ER oxidoreductase 1α, which regulates ER redox status, is upregulated with PERK signaling. The expression of ER oxidoreductase 1α is significantly increased in various types of cancer [67]. The PERK and ISR signaling pathways may be useful therapeutic targets for cancer. The PERK-specific inhibitor GSK2656157 was reported to inhibit angiogenesis and amino acid metabolism, thus preventing tumorigenesis in vivo [62].

A lack of PERK in MAMs will lead to ER breakage and abnormal calcium release. This functional change in the ER occurs because of the lack of PERK in MAMs. PERK activities have a variety of functions in the ER and MAM. Studies showed that PERK is involved in the adaptation of cancer cells to the challenges of the tumor microenvironment [68–70]. Some studies reported the presence of PERK in the ER in tumor cells, but the role of MAM-resident PERK remains unclear [70, 71]. Therefore, MAM-resident PERK may have pathological functions and be a therapeutic target of cancer.

### Phospholipid synthesis and transfer between mitochondria and ER

Phospholipids are a major component of all cell membranes, and the ER is the main site of phospholipid synthesis in cells. Phospholipids are normally transported in vesicles to their destination after synthesis in the ER. However, for transport into the mitochondria, phospholipids are directly imported through the membranes [72, 73]. MAMs do not only control the lipid membrane homeostasis of mitochondria and ER but also support the transfer of different lipids and have biological effects on cell fate [74]. A large number of lipid-metabolizing enzymes are abundant in MAMs, where lipid metabolism is also performed [8] (Fig. 1). One manner in which tumor cells inhibit mitochondrial metabolism and apoptosis signals is to alter the ER lipid structure, thus destroying the normal MAM raft.

Phosphatidic acid is converted into phosphatidylserine (PS) in the ER, as the ER contains the relevant enzymes phosphatidylserine synthase 1 (PSS-1) and PSS-2 [75]. The IMM contains PH and SEC7 domain-containing protein 1 (PSD), which converts PS into phosphatidylethanolamine (PE). Therefore, PS must be transferred to the OMM and further transferred to the IMM, where it is converted into PE [75]. The rate-limiting step of PE generation is that PS enters mitochondria through MAMs [75]. Finally, PE returns to the ER, where phosphatidylethanolamine N-methyltransferase 2 (PEMT2) methylates PE to synthesize phosphatidylcholine (PC). However, as mitochondria also contain PC, PC is transferred from the ER into the mitochondria. Therefore, in order to achieve the final lipid composition of both organelles, a large amount of lipid exchange must be performed between these two organelles.

In addition, phosphatidic acid is an important source material for the synthesis of cardiolipin (CL). Phosphatidic acid is transferred from the ER to the OMM and then transferred to the IMM. It is converted into cytidine diphosphate diacylglycerol by the mitochondrial translocator assembly and maintenance protein 41 homolog (Tam41) in the IMM [76]. Next, cytidine diphosphate diacylglycerol further synthesizes glycerol-3-phosphate phosphatase with glyceraldehyde 3-phosphate under the catalysis of phosphatidylglycerophosphate synthase 1; glycerol-3-phosphate phosphatase is dephosphorylated by the phosphatase Gep4 to generate phosphatidylglycerol [77]. Although phosphatidylglycerol is only present in small quantities in the mitochondria, it plays an important role in CL synthesis [78], catalyzed by CL synthase Crd1 [79].

Analysis of the intimal lipid composition of various tumor mitochondria revealed that its cholesterol content was high, and changes in fatty acyl components were observed. Mitochondrial phospholipids in tumor cells are typically shorter than those in normal cells and unsaturated acyl chains are shorter [80, 81]. The composition and content of CL is significantly altered in some tumors [82], which is likely related to defects in CL synthesis and remodeling.

In addition, MAMs contain enzymes that are necessary for cholesterol and ceramide biosynthesis [83, 84]. In hepatocytes, acetyl-CoA acetyltransferase, mitochondrial ACAT1 in the MAM catalyzes the formation of cholesterol esters in the resting state, thereby controlling the balance between membranous and cytoplasmic lipids and low-fat cholesterol. In response to stress, cholesteryl esters are continuously introduced into the mitochondria, and cytochrome P450 initiates steroidogenesis [83]. Ceramide synthetized in the ER flows into the mitochondria and permeabilizes the OMM to apoptotic-inducing proteins, thus initiating apoptosis. MAMS can be considered a specific ceramide pool containing sphingomyelin phosphodiesterase (SMase), ceramide synthase (CerS), and dihydroceramide desaturase (DES). Considering the pro-apoptotic properties of ceramide in the mitochondria, MAM may act as an important reservoir or barrier to prevent the influx of ceramide into the mitochondria.

Cholesterol metabolism is deregulated in carcinogenesis, and cancer cells exhibit increased mitochondrial cholesterol content. Changes in mitochondrial cholesterol transport and metabolism in cancer cells affect the biophysical properties and mitochondrial functions of mitochondrial membranes. Compared to normal cells, the mitochondria of cancer cells are more susceptible to increased cholesterol, which triggers ER stress and apoptosis [85]. Ceramide is considered a tumor suppressor lipid because of its important role in regulating the physiological and drug-induced apoptosis of cells. The production of ceramide under the action of SMase was shown to be important in the regulation of cancer progression. Inhibition of SMase is related to drug resistance to a variety of anticancer drugs [86]. CerS expression was also shown to modulate the sensitivity to cancer chemotherapy drugs and radiotherapy. Overexpression of CerS1 in hek-293 cells was shown to make these cells sensitive to some anticancer drugs, such as cisplatin, carboplatin, doxorubicin, and vincristine.

## Mitochondria and peroxisomes

Peroxisomes are ubiquitous and dynamic single membrane-bound organelles in cells, who modulate their numbers, morphology, and activity to adapt to diverse environments in different tissues, organs, and nutritional states [87–89]. Peroxisomes play important roles in biosynthesis and signal transduction, including ether-phospholipid biosynthesis, fatty acid α-and β-oxidation, bile acid and docosahexaenoic acid synthesis, glyoxylate metabolism, amino acid catabolism, polyamine oxidation, the metabolism of reactive oxygen and nitrogen species, inflammation, innate immunity, and other processes, which cannot be achieved without interaction with other organelles in the cell [87, 88]. Among them, mitochondria and peroxisomes interact very closely. They cooperate with each other to maintain lipid balance through fatty acid β-oxidation, to maintain the balance of ROS in cells through scavenging, and to resist foreign invasion through antiviral reactions and other immune responses [87–89]. In this series of processes, mitochondria and peroxisomes can complete various biological functions through vesicles transport, signaling molecules, and membrane contact sites [90]. They also exhibit a close interplay in generation, fission, proliferation, and degradation [90]. The integrity and stability of peroxisomes are important guarantees for the maintenance of normal mitochondrial function. Peroxisomal dysfunction seriously affects mitochondrial metabolism, morphological stability, and biosynthesis, which directly or indirectly lead to rare genetic diseases, such as X-linked adrenoleukodystrophy, acatalasemia, and Zellweger syndrome, and relatively common age-related disorders, such as diabetes, neurodegenerative disease, and cancer [87, 91].

### The connections between the two organelles in biogenesis, degradation, and fission

**Fig. 2:**
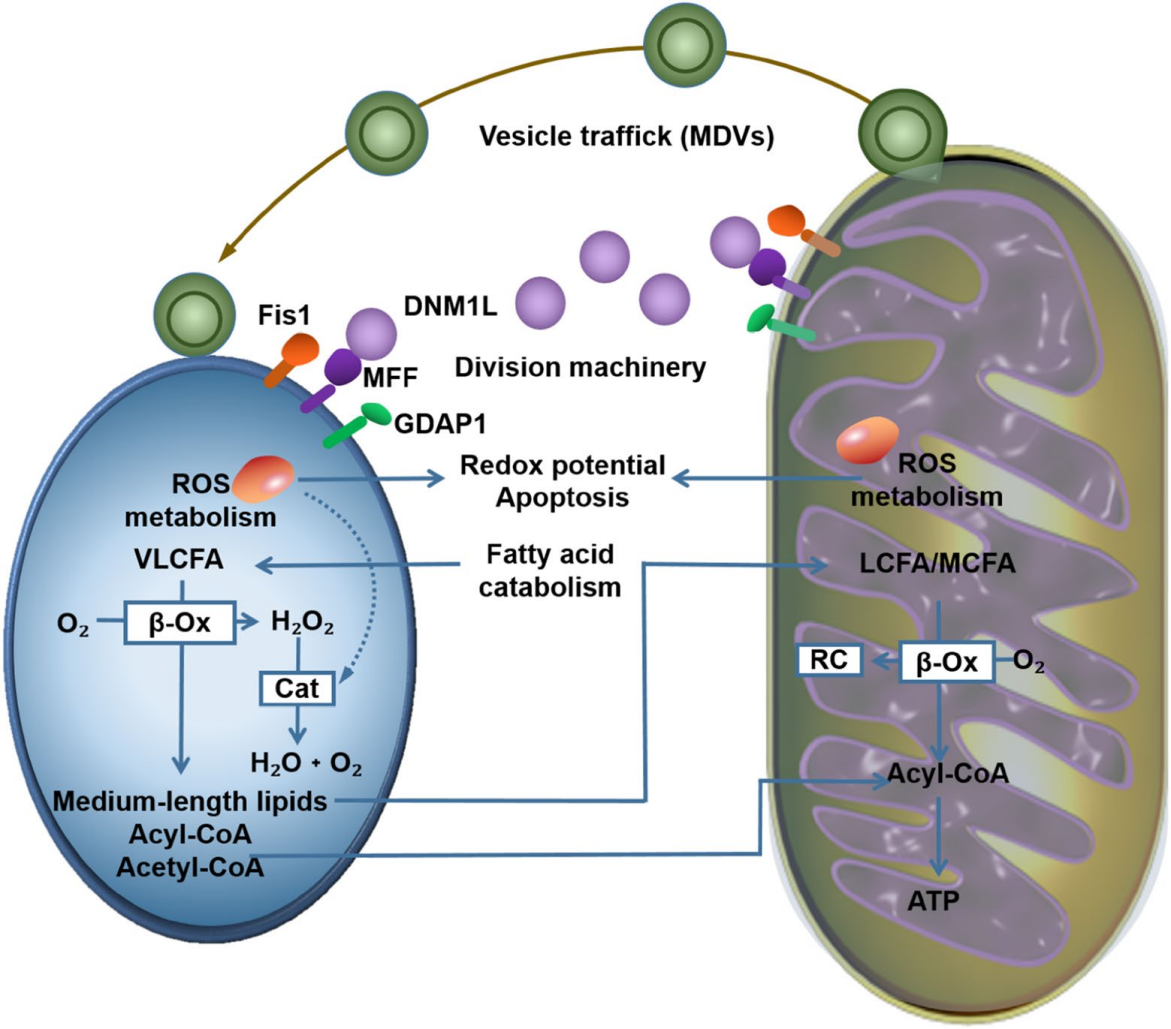
The connection between peroxisomes and mitochondria. Mitochondria can communicate with peroxisomes via vesicular transport of MDVs. Key fission components FIS1, MFF, and GDAP1 are shared by both peroxisomes and mitochondria, and they recruit DNM1L to the organelle cleavage site to disrupt organelles. The fatty acid β-oxidation can occur both in mitochondria and peroxisomes. However, the lipid β-oxidation in peroxisomes is not complete, after degrading lipids to medium length, they will be co-transported with acetyl-CoA to the mitochondria for further metabolism. Both mitochondria and peroxisomes can produce ROS, and they are also important organelles for removing ROS and ensuring cell stability. Peroxisomes mainly contain catalase to break down H_2_O_2_. ROS are also important signaling molecules, which can induce cell apoptosis

Mitochondria participate in the formation of peroxisomes. In mammals, peroxisomes can be produced by asymmetric growth and division from pre-existing organelles, as well as by the fusion of pre-peroxisomes from the ER and mitochondria [87, 91], allowing the transport of functional proteins and other compounds from the mitochondria into peroxisomes, which may be one of the reasons why peroxisomes and mitochondria have many similar functions [91] (Fig. 2).

The function of mitochondrial and peroxisomal coordination cannot be separated from the transcriptional regulation mechanism, including peroxisome proliferator-activated receptors (PPARs), whose different subtypes have different tissue expression patterns and substrate specificities as well as regulate different target genes [91, 92]. PPARs form a sub-family of nuclear hormone receptors that function as ligand-activated transcription factors to regulate various biological processes [93]. They can regulate the metabolism of cell lipids and carbohydrates, cell differentiation, and tumorigenesis after their activation by ligands that regulate the proliferation of mitochondria and peroxisomes and the expression of lipid β-oxidation related genes [92, 94]. The activity of PPARs is also regulated by many transcriptional coactivators and co-repressors [87, 91]. One coactivator is peroxisome proliferator-activated receptor gamma coactivator-1α (PGC-1α), which is a powerful transcriptional coactivator that modulates physiological and energy homeostatic responses at the transcriptional level in different mammalian tissues and can interact with nuclear receptors to enhance peroxisomal activity and the expression of mitochondrial biosynthetic factors, oxidative phosphorylation subunits, and antioxidant enzymes [95–97]. PGC-1α also promotes the generation of peroxisomes in the liver, muscle, and adipose tissues, independently of the effects of PPARs [87, 91]. However, PGC-1α can also induce the generation of peroxisomes in a PPARα-independent manner [97]. In summary, the abundance and activity of mitochondria and peroxisomes are regulated through PPARs and PGC-1α at the transcriptional level (Table 2).

**Table 2:** The function of related molecules of mitochondria and peroxisomes

Molecules	Function	References
PPARs	Regulate the metabolism of lipids and carbohydrates, cell differentiation, tumorigenesis, the proliferation of mitochondria and peroxisomes and the expression of lipid β-oxidation related genes	[92, 94]
PGC-1α	The generation of peroxisomes, expression of mitochondrial biosynthetic factors, oxidative phosphorylation subunits, antioxidant enzymes and unregulated in tumor cells in majority situation	[95–97, 114–116]
FIS1, MFF, GDAP1 and DNM1L	Fission and generation of peroxisomes and mitochondria	[87, 98, 99]

Both peroxisomes and mitochondria can be generated by fission from pre-existing organelles and share many proteins involved in division [98]. Mitochondrial fission 1 (FIS1) protein, mitochondrial fission factor (MFF), and ganglioside-induced differentiation-associated protein (GDAP) 1, membrane adapter proteins located on the mitochondria and peroxisomes membranes recruit dynamin-1-like protein (DNM1L) to the organelle cleavage site to disrupt organelles through a series of downstream post-transcriptional modifications. Overexpression or downregulation of membrane adaptor proteins induces splitting or elongation these two organelles, respectively [91, 98, 99] (Fig. 2, Table 2).

Dysfunctional and impaired peroxisomes in cells can be cleared by the lysosomal autophagy pathway called pexophagy [100]. The mitochondria are cleared by mutual fusion and phagocytosis by lysosomes called mitophagy [100]. A number of studies have shown that when the peroxisomal function is impaired, mitochondria can exert compensatory effects by increasing their volume through autophagy, but the specific molecular mechanism is not yet clear [91].

### The communication mechanisms between the two organelles

Mitochondria and peroxisomes are closely linked through membrane contact sites. In the past, researchers verified the close relationship between the two organelles by studying their spatial structure by using a series of experimental methods [88, 101]. In mammalian cells, mitochondria and peroxisomes contact each other through a complex whose core component is a splice variant of enoyl-CoA isomerase 2, which contains the targeting signals to mediate the close contact between the two organelles [91]. In yeast, peroxin-11, the most abundant peroxisomal membrane protein, is involved in peroxisome generation and composition, which regulates the division of peroxisomal membranes during proliferation [102]. The ER-mitochondrial encounter structure (ERMES) complex serves as a bridge between molecular exchanges and tight links of mitochondria and ER, whereas peroxin-11 binds to the mitochondrial component Mdm34 of the ERMES complex to mediate and promote information transfer between mitochondria and peroxisomes [102] (Fig. 2).

Mitochondria can also communicate with peroxisomes via vesicular transport of mitochondria-derived vesicles [98]. Among them, mitochondrial anchored protein ligase promotes the division of mitochondrial membrane and leads to the formation of vesicles [98, 99]. Next, the mitochondrial vesicles with mitochondrial anchored protein ligase fuse with peroxisomes. This fusion promotes the production of peroxisomes and transports certain specific metabolites and needed proteins to the peroxisomes [98, 99]. In addition, mitochondria and peroxisomes can be linked by the release of biological messengers, including ROS, lipids, or other metabolites, and this process is closely related to the size of the molecules and the permeability of the organelle membranes [87] (Fig. 2).

### The metabolic interplay between the two organelles

The β-oxidation of fatty acids occurs simultaneously in the mitochondria and peroxisomes, and each of the β-oxidation cycles involves four consecutive reactions [99]. In these two organelles, the enzymes used for the β-oxidation reaction are different and have different substrate specificities [87]. Dietary fatty acids such as palmitic acid, oleic acid, and linoleic acid are preferentially metabolized in mitochondria, and most carboxylic acid esters such as very-long-chain fatty acids, pristanic acid, other 2-methyl-branched prostanoids, and bile acid intermediates are more likely to be metabolized in peroxisomes [87, 91]. The lipid β-oxidation in peroxisomes is not complete and after oxidizing degradation of lipids to medium length, they are co-transported with oxidatively produced acetyl-CoA to the mitochondria for further metabolism [98, 99]. The acetyl-CoA is used to generate energy in the tricarboxylic acid cycle, and lipids in the mitochondria eventually produce CO_2_ and H_2_O [98, 99] (Fig. 2).

Both during the production of ATP in mitochondria and lipid β-oxidation in peroxisomes, ROS is produced, but mitochondria and peroxisomes are also important organelles in removing ROS and ensuring cell stability [103–105]. Peroxisomes mainly contain catalase to break down H_2_O_2_. When catalase function is altered or its production in peroxisomes is disturbed, it will lead to mitochondrial oxidative stress response and, in severe cases, IMM structure alteration, changes in respiratory chain complex activity, DNA damage, and increased organelle volume, which can further cause oxidative stress damage to the entire cell [105, 106]. Studies have shown that when ROS in peroxisomes exceeds a certain level, ROS level in mitochondria increases, and the redox balance in mitochondria is disturbed, causing mitochondrial breakdown and cell death [104–106]. In addition, ROS are important signaling molecules in cells, which can cause mitochondrial and peroxisomal autophagy and apoptosis [107]. When ROS level increases in cells to induce oxidative stress, the expression of starvation-induced protein DEPP is upregulated, which further induces autophagy, thereby protecting cells from injury [108]. Although how mitochondria and peroxisomes communicate through ROS has not been elucidated in detail, it is possibly through intracellular diffusion, potential contact sites, or vesicle trafficking [101] (Fig. 2).

### The link between ROS and cancer

Mitochondria and peroxisomes are important organelles in the production and clearance of ROS. Impaired peroxisomal function inevitably leads to an increase in ROS level in mitochondria, which damages the mitochondria and aggravates ROS clearance disorders, thereby promoting the occurrence and development of tumors [103] (Table 2).

ROS as signaling molecules can regulate a variety of physiological and pathological processes [109]. H_2_O_2_ is an ROS family member that plays an important role in the signal transduction process of epidermal growth factor (EGF) and platelet-derived growth factor (PDGF). H_2_O_2_ can prevent protein-tyrosine phosphatase 1B (PTP1B) from dephosphorylating EGF, thereby promoting EGF stimulation. Additionally, activation of PDGF requires H_2_O_2_ to facilitate PDGF-receptor-associated phosphatase and SHP-2 oxidation and inactivation, thereby promoting the signal pathway [110, 111]. PTEN is a negative regulator of the phosphoinositide 3-kinase (PI3K) signaling pathway and a tumor suppressor. Through oxidation and other effects of H_2_O_2_, PTEN inhibits the tumor suppressor function and promotes tumor proliferation [110]. ROS production promotes the genomic and chromosomal instability of the cells and mutations in the mitochondrial genome, promoting the production of ROS and signaling pathways in tumors such as PI3K and mitogen-activated protein kinase (MAPK) [110].

ROS production can promote the proliferation and survival of tumor cells under hypoxia conditions. In the absence of oxygen in tumor cells, hypoxia-inducible factors (HIFs), which are transcription factors, are upregulated to promote the expression of oncogenes. Some enzymes, such as prolyl hydroxylases (PHDs), cause degradation of HIFs. However, hypoxia induces an increase in ROS production, preventing the action of PHDs on HIFs, enabling HIFs to promote tumor development [110, 111].

However, ROS function in both normal cells and tumor cells. Some ROS are used as signaling molecules to activate intracellular autophagy and apoptosis pathways, whereas excessive ROS induce cell oxidative stress damage and eventually cell death [112, 113].

In tumor cells, increased metabolic rate, mitochondrial dysfunction, effects of oncogenes, and enhanced intracellular signal transduction lead to increased production of ROS [112]. On the one hand, this increase upregulates the antioxidant system to maintain the stability of the redox reaction in tumor cells; on the other hand, it promotes the development of tumors [112]. ROS promotes cellular DNA damage and genomic instability, and an increase in gene mutation rate easily leads to a malignant phenotype of cells [113]. ROS also induces mitochondrial gene damage and mutation, which further promote tumorigenesis [113]. Besides, ROS further contributes to the proliferation of tumor cells by promoting the function of growth factors and signal transduction [113]. Angiogenesis, invasion, and migration are the final stages of malignant transformation of tumor cells, and ROS promotes tumor angiogenesis by enhancing the expression and activity of vascular endothelial growth factor and hypoxia inducible factor 1α to provide oxygen and nutrition [113]. ROS also mediates the activity of matrix metalloproteinases and Wnt/β-catenin signaling pathways to promote invasion and migration of tumor cells [113], which provides ideas for clinical treatment, such as, reducing ROS production in tumor cells through a series of methods to suppress the occurrence and development of tumors.

However, when the production of ROS in tumor cells exceeds a certain limit, it becomes cytotoxic and reverses the ability of tumor cells to be resistant to chemotherapy [113]. ROS also upregulates the activity of caspase family proteins and death receptor 5 to promote apoptosis, which eventually leads to cell death [113]. A number of studies have shown that PGC1α is upregulated in a variety of tumor cells, which promotes mitochondrial production and the expression of enzymes involved in mitochondrial metabolism [95, 96, 114, 115]. On the one hand, PGC1α can provide more energy for tumor cells by promoting the process of oxidative phosphorylation and the tricarboxylic acid cycle, making tumor cells metabolically superior to normal cells; on the other hand, it can enhance the clearance ability of ROS in tumor cells to protect them from oxidative stress [114–116]. However, there are also other reports in the literature that show that the expression of PGC1α is downregulated in some tumors, which may be related to the growth stage and the metabolic conditions of tumor cells [117, 118]. Therefore, upregulation of ROS by specific treatments in tumor cells can also inhibit the tumor growth.

### Deficiency and prospects

The interaction between mitochondria and peroxisomes is an important part of maintaining cell stability, and plays an extremely important role in cell metabolism, biosynthesis, and cell fate. Meanwhile, their communication also has an important effect on immune response and resistance of virus infection in host cells. However, many processes have not been researched in detail. For example, the specific details and mechanisms of how mitochondria and peroxisomes communicate through signaling molecules and metabolites, how mitochondrial dysfunction affects peroxisomes and the specific mechanisms of how peroxisome damage affects the mitochondria, and the details of the relationship between mitochondria and peroxisomes in generation are not known. Alteration in the ROS level in peroxisomes will rapidly change the levels in mitochondria; however, how the two interact with each other via a redox mechanism is also unclear. At the same time, in terms of tumor therapy, ROS boosting or ROS scavenging can be applied to the clinic because too much or too little ROS can affect the occurrence and development of tumors. Nowadays, changing ROS level in cells by using pro-oxidants and antioxidants plays an important role in the clinical treatment of tumors cells [113]. Tumor cells have regulatory mechanisms that can adapt to the alteration of redox balance, such as those involving PPARs and PGC-1α, which regulate mitochondria and peroxisomes at the transcriptional level. Effectively destroying this regulatory mechanism will facilitate the treatment of tumors. Therefore, understanding the precise regulatory mechanism between the mitochondria and peroxisomes is the next goal.

## Mitochondria and nucleus

As the only organelle possessing an independent genome in the eukaryotic cell, the mitochondrion has its own lifespan in the cell cycle [119, 120]; however, among the more than 1200 mitochondrial proteins, only 13 are encoded by the mitochondrion itself, and most of the mitochondrial proteins are encoded by the nucleus [121]. The mitochondria biogenesis are modulated by the nucleus genome and the mitochondria genome, Therefore, there is a precise and strict regulatory mechanism between the nucleus and mitochondria to control the stability of mitochondria. A dysfunctional crosstalk between these two organelles leads to DNA damage in both the nucleus and mitochondria, calcium overload, and abnormal activation of growth factors [121], as well as metabolism disorders that are hallmarks of carcinoma [122].

### Anterograde regulation

**Fig. 3:**
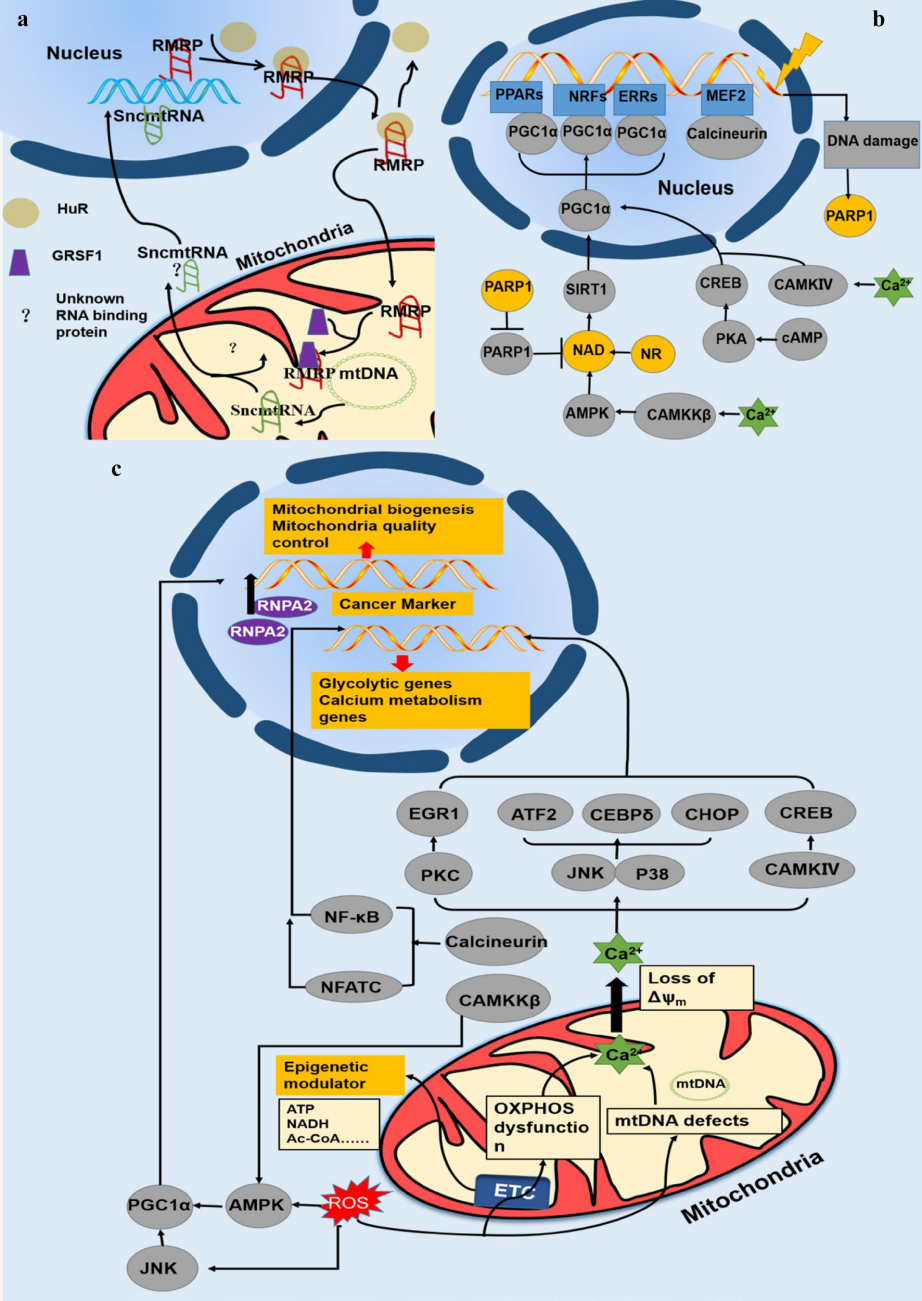
The mitonuclear communication. **a** Noncoding RNA in nucleus-mitochondria regulation (using lnc RMRP as an example). The lnc RMRP transcribed in nucleus is translocated in the mitochondria and targets the mtDNA, while the mtDNA encoding small noncoding RNA (snmtRNA) can be transferred to the nucleus. Both processes require the cooperation of RNA-binding proteins such as HuR and GRSF1. **b** Anterograde regulation. Different cytoplasmic stressors initiate several signaling pathways and activate the same co-activator PGC1α, which subsequently stimulates different transcription factors and nuclear receptors as shown in Fig. 3. The downstream signaling of these transcription factors and nuclear receptors regulates mitochondrial biogenesis. Not all transcription factors, co-activators, and co-repressors are shown in Fig. 3 (see Refs [119, 120]). **c** Retrograde regulation. Retrograde signaling is triggered by OXPHOS dysfunction and mtDNA defects, which result in the loss of mitochondria potential. The retrograde pathway involves Cn-calcium signaling, AMPK signaling, and activation of molecules such as HnRNP A2, which is a cancer hallmark. Retrograde regulation also increases the expression of certain epigenetic modulators that regulate the nuclear epigenome

The nucleus controls the proteins and information transmitted to the mitochondria by anterograde regulation. Anterograde regulation reflects different stressors through the nuclear genome reprograming which modulate mitochondria biogenesis. The transcription of multiple mitochondrial proteins requires a simple RNA polymerase (POLRMT) and mtDNA transcription factor-Tfam1, Tfb1 m, Tfb2 m, and transcription termination factor (MTERF). Transcriptional control in the mitochondria involves multiple transcription factors and co-activators. Anterograde regulation mainly depends on two set of factors; the first is nuclear respiratory factor 1 (NRF1) and NRF2 which modulate OXPHO genes and mitochondria DNA replication and expression, with NRF1 playing a leading role in this process [123, 124]. By binding to the cytochrome C promoter, NRF1 directly or indirectly regulates mitochondria biogenesis by activating genes related to OXPHO or decreasing other transcription factors such as MEF2A which is related to mitochondria biogenesis. The second set of factors is the PGC family containing PGC1α, PGC1β, and PGC1-like factor, PRC. PGC functions as a co-activator by integrating all physiologic signals and enhancing the function of other transcription factors [125, 126] (Fig. 3c).

Nuclear receptors such as PPARs or estrogen-related receptor (ERR) can also initiate anterograde regulation. PPARδ stimulates enzymes involved in fatty acid oxidation, particularly in the heart and muscle tissue, and ERRs modulate the expression of nuclear-encoded-protein in TCA cycle, oxidative phosphorylation (OXPHOS), and the fatty acid oxidation process. The finely tuned regulation of transcription also involves other transcription factors such as CREB, c-myc, and YY1 and co-repressors such as receptor interacting protein 140 (RIP140) to maintain the balance with co-activators. Additionally, chemical modification such as phosphorylation of the transcription factors and/or co-activator/co-repressor occurs during anterograde regulation [127, 128] (Fig. 3c).

The transcriptional control of mitochondria biogenesis is tissue- and organ-specific and different stressors initiate different downstream pathways.

When cells are under stress such as calcium overloading, oxidative pressure, and DNA damage, different stressors activate different downstream cascades, resulting in the activation of different cascades. For example, when ATP production decreases, as observed following exercise and caloric restriction, the rate of AMP/ATP is increased, upregulating the downstream molecules NAD^+^, sirtuin-1, and PGC1α and promoting mitochondria energy metabolism and biogenesis, whereas calcium overload activates calcium/calmodulin dependent protein kinase type IV or 5′ adenosine monophosphate-activated protein kinase (AMPK) and promote mitochondria biogenesis [119, 129]. By activating specific transcription factors, the nucleus maintains homeostasis in the mitochondria under stress conditions (Fig. 3c).

In addition to activation of multiple stressors, nuclear DNA damage may initiate anterograde regulation. A study showed that nuclear DNA damage triggering anterograde regulation are indispensable in aging [130], which directly upregulates the rate of aging-associated diseases such as neurodegeneration and cancer. The transcriptional alteration of mitochondrial proteins may lead to severe disorders, such as osteosarcoma, breast cancer, and prostate cancer. However, the specific mechanism of anterograde signaling in tumorigenesis has not been widely studied, in contrast to retrograde signaling discussed below.

### Retrograde regulation

To maintain a tight cooperation between the mitochondria and nucleus, the mitochondria may also regulate the function of nucleus through retrograde regulation. Loss of the mitochondria membrane potential in the mitochondrial respiratory chain disorders, by DNA mutation or change of the mitochondrial DNA copies [131], initiate the retrograde regulation from mitochondria to the cell nucleus. Many processes in retrograde regulation are considered as markers in tumorigenesis. For example, mtDNA alterations are common in tumorigenesis, and the accumulation of heterogeneous nuclear ribonucleoprotein A2 is a hallmark of cancer. The first confirmed retrograde regulation pathway was found in *Saccharomyces cerevisiae* and called the RTG-dependent pathway, which involves Rtg1p, Rtg2p, and Rtg3p [132, 133]. When the Rtg1p/Rtg3p complex is activated, it translocates into the nucleus and affects gene transcription. Although only Rtg3p possess a DNA-binding site, the formation of the Rtg1p/Rtg3p complex is still required for the whole process. This process has also a connection with the mammalian target of rapamycin (mTOR) pathway. McKusick-Kaufman syndrome 1 binds to Rtg2, freeing the structural maintenance of 14-3-3 family protein BMH1/2 [134], which removes the inhibition of the Rtg1p/Rtg3p complex. Another study suggest that G protein pathway suppressor 2 may act as Rtg2 in mammalian cells by avoiding the methylation of histone H3k9 [135].

In mammals, retrograde regulation clearly involves the mTOR/AMPK pathway and the mitochondria calcium signaling. As mitochondria are important in calcium buffering, Calcium flows into the cytoplasm from the mitochondria when the mitochondrial function is altered, causing a loss of membrane potential and activating phosphatase calcineurin, which in turn affects gene transcription through nuclear factor-κB and nuclear factor of activated T-cells (Fig. 3b).

Also, the mitochondrial DNA is near to the ROS generation site and has a less sophisticated recovery mechanism, which leads to the mitochondria DNA being more vulnerable under oxidative stress (Fig. 3b).

The mitochondrion OXPHOS and TCA cycle involves genes that easily get damaged in tumorigenesis; however, cancer cells still rely on energy supplied by the mitochondria, in which retrograde signaling plays an important role [1]. Defects in succinate dehydrogenase (SDH) increase succinate levels in the mitochondria, initiating HIF-α signaling and shifting the cell metabolism model from OXPHOS to glycolysis [1, 136]. Defects in fumarate hydratase (FH) increase fumarate levels in mitochondria, which activates NRF2 signaling and increases the expression of heme oxygenase (HMOX1), which is beneficial for forming colonies [1, 137, 138]. Isocitrate dehydrogenase1 (IDH1) and IDH2 disturb the redox status of important transcription factors by preventing the reaction of isocitrate to α-ketoglutarate to stimulate cell proliferation and tumorigenesis [139, 140]. Mutation of all four genes may lead to changes in chromatin methylation and epigenetic modification (Table 3).

**Table 3:** Retrograde signaling in tumorigenesis

Triggers	Pathway	Function	References
mtDNA defect	Cn-calcium path way	EMT-like reprograming	[143]
	Epigenetic modification	Unclear	[121, 141]
Mitochondria metabolism disorder	HIF-α	Metabolism model shift	[136]
	NRF2	Cell colony capacity	[1, 137, 138]
	Mitochondria redox signaling	Cell proliferation	[139, 140]
mtDNA defect/mitochondria metabolism disorder	Activation of ocogenic kinase	Cancer hallmark	[121]
	HnRNP A2 accumulation		

Moreover, the mechanism of the epigenetic modification triggered by the mtDNA mutation and alteration of mtDNA copy also connect to some cell metabolism products. The mitochondria satisfy the cell energy demand through the TCA cycle, fatty acid oxidation, and the electron transfer complex, which convert fatty acid and glucose into NADH, acetyl-DNA, and ATP, which can be messenger molecules in modulating the epigenetic modification of the nuclear genome. Therefore, the mitochondria can be an important junction connecting the cell metabolism and cell genome epigenetic modification by regulating the expression of ATP, NAD/NADH, and acetyl-CoA, which are capable of facilitating or inhibiting gene transcription. Studies also show that the mitochondria can influence DNA methylation by regulating the S-adenosyl methionine [121, 141] (Table 3, Fig. 3b).

More importantly, the mtDNA depletion-induced activation of calcium and phosphatase calcineurin (Cn) in the retrograde regulation pathway of mitochondria can lead to genetic and epigenetic changes that are beneficial for apoptosis resistance. The Cn-dependent pathway also participates in epithelial–mesenchymal transition (EMT) in breast cancer and it is induced by the copy number variation of the mitochondrial DNA [142]. EMT is regarded as the major regulator of metastasis, and the reduced mtDNA in human mammary epithelium cell(hMECs) can initiate Cn-dependent mitochondrial retrograde regulation and cause cell polarity loss, which seems like an EMT-like reprogramming, facilitating tumor invasion and migration [143] (Table 3, Fig. 3b).

### Noncoding RNA in the communication between the nucleus and mitochondria

Intriguingly, researchers have recently highlighted the important role of noncoding RNA in the communication between the nucleus and mitochondria, as the noncoding RNA nearly covers the whole process of gene transcription and expression. Besides the anterograde and retrograde regulation reviewed above, a study uncovered putative small noncoding RNA encoded by mitochondria genome through the analysis of a small noncoding RNA library of *Ruditapes philippinarum.* The structure of those small noncoding RNA are close to the microRNA so that the researchers wonder that if the putative small noncoding RNA serve as a potential regulator of the nuclear functionthrough a microRNA-like mechanism [144, 145] (Fig. 3a).

The theory of noncoding RNA as a new regulator in nuclear and mitochondria communication is beginning to emerge; most studies have focused on long noncoding RNA (lncRNA). LncRNA regulates nuclear and mitochondria communication. Nuclear-encoded lncRNA are transmitted into the mitochondria and coordinate mitochondria-induced apoptosis [146, 147], mitochondria metabolism, and mitochondria biogenesis. For instance, ENS-MUST00000136025 stimulates the Bim gene, in turn coordinating mitochondria-induced apoptosis and lncRNA MEG3-induced apoptosis in renal cells by activating the mitochondria pathway [146]. In contrast, lncRNA encoded by the mitochondria modulate nuclear genome reprogramming. The mitochondria genome-encoded lncRNAs are classified into three groups: (1) lnc5, lnc6; (2) chimeric mitochondria-encoded lnRNA; (3) putative mitochondria-encoded lnRNA. Propagation of the lncRNA from the nucleus to the mitochondria requires the cooperation of RNA-binding proteins such as human antigen R protein (HuR), G-rich RNA sequence-binding factor 1 (GRSF1), and SMRT/HDAC-associated repressor protein (SHARP) [148]; however, the specific mechanism of the transportation of noncoding RNA from the nucleus to the mitochondria is unclear (Fig. 3a).

MicroRNAs are also vital in the interplay between the two organelles. For example, miR181c directly enters the mitochondria and affects the transcription of its target gene, while miR663 affects OXPHOS in the mitochondria and accelerates carcinoma by downregulating the expression of ubiquinol-cytochrome c reductase complex assembly factor 2 [149]. has-miR4485 is transcribed in the nucleus and resides in the mitochondria; this miRNA binds mitochondria 16 s rRNA to reduce tumorigenesis in breast cancer cells (Table 4).

**Table 4:** Noncoding RNA in nuclei and mitochondria interaction

Genome source	Noncoding RNA	Interacting protein (for lncRNA)	Target (for miRNA)	Function references
*Mitochondrial DNA*	lncND5	MRPP1		Mitochondrial gene expression [147]
	lncND6			
	lncCyt b			
	SncmtRNA			Unknown [150]
	SmithRNA?	Unknown		Bending nuclear DNA? [145]
*Nuclear DNA*	miR-663		UQCC2	EMT [149]
	miR-4485		Mitochondria 16 s rRNA	Modulate mitochondria complex I [151]

The functions of noncoding RNAs in nuclear and mitochondria communication in tumorigenesis are unclear. Based on their specific mechanisms of action, further studies should focus on identifying the targets of certain noncoding RNAs to determine their roles in tumorigenesis.

## Conclusion

The mitochondria are the Achill’s hell of malignancy, especially when recent studies shows the complex connection between the mitochondria and cancer, especially the cancer metabolism. The cancer cells do not just rely on the dysfunction state of mitochondria like the “Warburg effect” suggest but manipulate the mitochondria and turn it into its energy factory through the mechanisms mentioned before, such as the HIF-αsignaling in the retrograde regulation. However, there are still numerous mystery of the role that mitochondria might play in tumorigenesis to be solved, for example, the specific mechanism of mtDNA mutation and defects in tumorigenesis. Despite the metabolism angle that we used to visualize the mitochondria in cancer, the organelle interplay may offer a new clue for further exploration in clinical drug design and development.

Currently, we study the cell as a whole and not as a mix of different organelles. Researchers are realizing that organelles are communicating and maintaining the cell homeostasis through their tight connection. It is universally acknowledged that the mitochondrion is the major source of energy, and its importance cannot be disputed. Therefore, it is indispensable for us to explore the interplay between the mitochondria and other organelles. As elaborated above in this review, understanding the communication between mitochondria and the ER, peroxisome and nucleus is necessary in terms of understanding the role that mitochondria play in tumorigenesis.

The mitochondria in cancer involves the pathways like the PI3 K pathway, P53 pathway, calcium related pathway etc. which are important in cell metabolism, cell proliferation, mitochondria induced cell apoptosis and so on. We regard the mitochondria a decent target in cancer treatment for the reason that the mitochondria is pivotal in the cell cycle.

Ongoing clinical trials and drug development are mainly focusing on the metabolism mechanism such as electron transport chain, TCA cycle and the Oxidative phosphorylation. Other drugs also target at the calcium buffering the signaling pathways involved in this communication.

The mitochondria are a rather multifunctional organelle; hence, considering a single mechanism or focusing on a single target can be counterproductive. For example, the use of pro-oxidants to destroy the redox balance in tumor cells promotes cell death, but it also increases the risk of normal cell canceration. The use of antioxidants reduces intracellular ROS in tumor cells, thereby weakening their mutation and invasive ability, but it also attenuates the ability of ROS to induce cell injury and apoptosis, which promotes tumor development to a certain degree.

However, studies on organelle communication are limited. Cancer exhibits great heterogeneity, and thus, it is unclear whether mechanisms involved in tumor cells occur in other tissues and/or organs. Moreover, recent studies have focused on the molecular level, and thus, further investigations are needed using animal models, which would be more significant for clinical application and drug development.

Therefore, considering the complexity of organelle interaction, there are still unknown molecular mechanisms that warrant further exploration.

## Abbreviations

TCA: tricarboxylic acid
MAMs: mitochondria-associated ER membranes
IP3R: inositol 1,4,5 trisphosphate receptor
VDACs: voltage-dependent anion channels
OMM: outer mitochondrial membrane
MCU: mitochondrial calcium uniporter
IMM: inner mitochondrial membrane
GRP-75: glucose regulated protein 75
MFN2: mitofusin 2
PTEN: phosphatase and tensin homolog on chromosome 10
PML: promyelocytic leukemia
Bcl2-L-10: Bcl-2-like protein 10
IRBIT: IP3Rs binding protein released with IP3
SERCA: sarco/endoplasmic reticulum calcium ATPase
STIM: stromal interaction molecule 1
SOCE: store-operated calcium entry
CRAC: calcium-released activated channels
UPR: unfolded protein response
PERK: RNA-dependent protein kinase-like kinase
ATF6: activating transcription factor 6
IRE1α: inositol-requiring enzyme 1α
eIF2α: eukaryotic translation initiation factor 2α
ISR: integrated stress response
PS: phosphatidylserine
PE: phosphatidylethanolamine
PC: phosphatidylcholine
CL: cardiolipin
PSD: PH and SEC7 domain-containing protein
SMase: sphingomyelin phosphodiesterase
CerS: ceramide synthase
DES: dihydroceramide desaturase
PPARs: peroxisome proliferator-activated receptors
PGC-1: αphosphatidylglycerol
FIS1: mitochondrial fission 1 protein
DNM1L: dynamin-1-like protein
GDAP: differentiation-associated protein
MFF: mitochondrial fission factor
ERMES: ER-mitochondrial encounter structure
EGF: epidermal growth factor
PDGF: platelet-derived growth factor
PTP1B: protein-tyrosine phosphatase 1B
PI3K: phosphoinositide 3-kinase
MAPK: mitogen-activated protein kinase
HIFs: hypoxia-inducible factors
PHDs: prolyl hydroxylases
OXPHOS: oxidative phosphorylation
snmtRNA: mtDNA encoded small noncoding RNA
POLRMT: RNA polymerase
MTERF: transcription termination factor
NRF1: nuclear respiratory factor 1
ERR: estrogen related receptor
RIP140: receptor interacting protein 140
AMPK: 5′ adenosine monophosphate-activated protein kinase
mTOR: mammalian target of rapamycin
Cn: calcineurin
SDH: succinate dehydrogenase
FH: fumarate hydratase
HMOX1: haem oxygenase I
DH1: isocitrate dehydrogenase 1
EMT: epithelial–mesenchymal transition
hMECs: human mammary epithelium cell
lncRNA: long noncoding RNA
HuR: human antigen R protein
GRSF1: G-rich RNA sequence-binding factor 1
SHARP: SMRT/HDAC associated repressor protein
